# Prompting Children’s Belief Revision About Balance Through Primary and Secondary Sources of Evidence

**DOI:** 10.3389/fpsyg.2020.01503

**Published:** 2020-07-22

**Authors:** Nicole E. Larsen, Vaunam P. Venkadasalam, Patricia A. Ganea

**Affiliations:** Department of Applied Psychology and Human Development, University of Toronto, Toronto, ON, Canada

**Keywords:** science learning, belief revision, balance, anomalous evidence, explanations, picture books, guided activity

## Abstract

Prior evidence has shown that children’s understanding of balance proceeds through stages. Children go from a stage where they lack a consistent theory (*No Theory*), to becoming *Center Theorists* at around age 6 (believing that all objects balance in their geometric center), to *Mass Theorists* at around age 8, when they begin to consider the distribution of objects’ mass. In this study we adapted prior testing paradigms to examine 5-year-olds’ understanding of balance and compared children’s learning about balance from evidence presented through primary sources (a guided activity) or secondary sources (picture books). Most of the research on young children’s understanding of balance has been conducted using a single object, weighted either proportionally (symmetrical object) or disproportionally (asymmetrical object). In this study, instead of using a single object, 5-year-olds (*N* = 102) were shown 4 pairs of objects, two with the same weight and two with different weight. Children were told to place the objects on a beam where they thought they would balance. We found evidence for an intermediate level of understanding. *Transition Theorists* represent children who have two distinct theories, one for balancing same weight objects, and one for balancing different weight objects, but one of these theories is incorrect. Following the assessment of children’s understanding, we compared their learning about balance from evidence that was either presented through primary sources (a guided activity) or secondary sources (picture books). Children learn equally well from both sources of evidence. Findings are discussed in terms of theoretical and practical implications.

## Introduction

Children build naive theories about the world around them and the physical rules that govern it through their daily first-hand observations and experiences (Brewer et al., 1998; Baillargeon, 2002). For some concepts children create beliefs that are counter to valid scientific conceptions. For instance, children may observe a bowling ball and a feather falling and may develop the incorrect idea that heavier objects fall faster than light ones. However, for other concepts, children’s conceptions may be correct, but partly incomplete (Clement, 2013). For instance, children learning to balance objects first form the belief that objects balance at their geometric center. This is true only for objects with an evenly distributed mass and represents a partial understanding of balance principles. Revising prior beliefs can be difficult because naive theories are built on the basis of first-hand observations and can also be driven by cognitive biases (Karmiloff-Smith and Inhelder, 1974; Shtulman, 2017; Kelemen, 2019). Also, just like adults, children easily interpret events that support a theory, but treat counterevidence (i.e., anomalies) as exemptions and isolated cases from their current theory (Karmiloff-Smith and Inhelder, 1974; Chinn and Brewer, 1993). Even when they consider the counterevidence, they tend to create a new theory to account for it, independent of their existing theory (Karmiloff-Smith and Inhelder, 1974). The current study examines children’s belief revision about balance relations. Specifically, we investigated how children’s beliefs about balance are impacted when the evidence is presented through primary sources (guided activity) or secondary sources (picture books). We also developed a new framework for characterizing children’s theories about balance that accounts for intermediate levels of understanding.

### Balance Literature

Beginning with the pioneering work of Karmiloff-Smith and Inhelder (1974), research has focused on three theoretical phases that children go through as they develop an understanding of balance. Children younger than six generally balance both symmetrical and asymmetrical blocks using a system of trial and error, with no clear reasoning behind their choices (Karmiloff-Smith and Inhelder, 1974), and so they are usually considered in the “No Theory” stage. Around the age of six, children become “Center Theorists” and begin to balance all blocks using the geometric center of an object as their reference point (Karmiloff-Smith and Inhelder, 1974). That is, when given a single block to balance, they place the geometric center on the fulcrum, as opposed to the center of mass, regardless of whether they are balancing symmetric or asymmetric blocks. Around the age of seven or eight, children begin to consider the distribution of weight, thereby using the center of mass as the balance point (Karmiloff-Smith and Inhelder, 1974). “Mass Theorists” can correctly balance both symmetrical and asymmetrical blocks.

Research by Siegler (1976) has also shown that children use four rules as they build an accurate understanding of the concept of balance. Children go from focusing on the relative weight on the two sides of the fulcrum (Rule 1), to focusing on the distance from the fulcrum if there are equal weights on both sides, but only on the relative weight when the weights are unequal (Rule 2), to an understanding based on both weight and distance for equal and unequal weights (Rule 3), and finally to an understanding based on computing torques on both sides (Rule 4). In contrast to the task used by Karmiloff-Smith and Inhelder (1974), in which children had to balance symmetrical and asymmetrical blocks, Siegler (1976) showed children a scale that had either equal and unequal weights placed at various distances from the fulcrum and asked them to predict whether the scale would balance. In this task, 4 and 5-year-old children used weight to inform their predictions (Siegler and Chen, 1998), indicating the presence of an incipient theory of balance.

Though Siegler’s balance rules have expanded on the theoretical phases put forth by Karmiloff-Smith and Inhelder, recent developmental research investigating young children’s understanding of balance relations has primarily used the placement task developed by the latter (e.g., Bonawitz et al., 2012; Ganea et al., 2017). However, because this task uses a single block, results map either onto Center Theorists (Rule 1 of Siegler’s model) or Mass Theorists (Rule 3), without allowing for consideration of an intermediate phase (Rule 2). In this research we combined both tasks to examine whether 5-year-old children hold a hybrid theory for balancing objects. In addition, we aimed to determine whether children’s belief revision is influenced by how the evidence is presented.

### Evidence Evaluation

Our ability to process and make judgments about evidence is highly influenced by prior beliefs, insofar as we are inclined to draw conclusions that are in line with these beliefs rather than to change them (e.g., Kuhn et al., 1988; Dunbar and Klahr, 1989; Kuhn, 1989; Schauble, 1990; Chinn and Brewer, 1993; Koslowski, 1996; Zimmerman and Klahr, 2018). The process of belief revision can be particularly difficult for children, as they need to accommodate not only the pull of naïve prior beliefs, but the cognitive demands of evidence evaluation as well (Tolmie et al., 2016). Evidence evaluation requires children to be able to track, connect, and remember different instances of similar phenomena; to understand that variables operate in a consistent fashion across said phenomena; and to connect these instances to their theories. Given these high demands on cognitive load, children may fail to engage in belief revision due to the complexity of the task, or because the evidence is not salient enough (Koerber et al., 2017). Demands on cognitive load also come from having to consider multiple variables in the interpretation of anomalous evidence, as is the case for balance (Siegler, 1976). Given these inherent difficulties, an important consideration is how to support children’s ability to learn from evidence.

One way to improve learning from anomalous evidence is to provide a plausible explanation that can explain the evidence. An explanation can reduce the bias toward maintaining prior beliefs in the face of counterevidence. In the absence of such an explanation, people are more likely to cling to their prior beliefs (Klahr and Dunbar, 1988; Kuhn, 1989; Chinn and Brewer, 1993). That is, people often consider an inadequate theory as preferable to the absence of a theory. Therefore, combining explanations with anomalous evidence may be the most effective way to promote theory change. There is evidence that children find claims more believable when they can generate, or are provided with, a possible mechanism to explain anomalies (Sandoval et al., 2014). Moreover, children are better able to revise prior beliefs when the evidence is linked to their current beliefs directly (Hardy et al., 2006) and when alternative theories are given prior to the counterevidence (Renken and Nunez, 2010, 2013; Ganea et al., 2020).

Another teaching strategy for increasing children’s learning in domains where they have misconceptions involves the use of multiple examples or analogies (Vosniadou, 1989; Vosniadou and Skopeliti, 2019). The use of analogies through the comparison of multiple examples can lead one to infer the deep abstract features that characterize a concept (Gentner, 1983, 1989) and allows for better visualization of scientific explanations (Vosniadou, 1989; Brown, 1993). In work with third-, fifth-, and sixth-grade children, as well as college students, Vosniadou and Skopeliti (2019) found that text with relevant analogical examples can reduce invalid inferences and misconceptions in understanding of the day/night cycle and seasons. However, work by Brown (1992) found that examples and analogies alone are ineffective when the target problem is in a domain where students hold misconceptions. This may be because students’ pre-existing conceptions interfere with the extraction of an abstract schema that accounts for the multiple examples. An effective way to improve this schema abstraction is by adding explanations, which help with the development of analogical connections (Brown, 1992). In this research, we use both examples and explanations to examine children’s learning about balance relations.

### Sources of Evidence

How evidence is presented to children can also have an impact on their learning. Evidence could be experienced either directly (primary evidence) or indirectly (secondary sources). In science, primary evidence refers to data that is derived from direct experience with an event or object (Sandoval et al., 2014). By extension, learning through primary sources of evidence involves children conducting or witnessing an experiment firsthand. For many concepts, however, primary evidence is inaccessible to the majority of people. Indeed, people are more often forced to consider and evaluate claims without access to such primary evidence, having instead to rely on secondary sources of evidence (Sandoval et al., 2014). Secondary sources of evidence provide an account of primary source evidence through various mediums such as books, websites, or verbal testimony.

Sandoval and Çam (2011) found that third and fourth grade students preferred primary data over other sources, citing the improved credibility of this evidence. Further, second grade students who were asked to evaluate a claim mainly used the primary evidence available to them, ignoring the secondary information (Koerber et al., 2017). Nevertheless, a related body of research indicates that young children have the capacity to learn from secondary sources of evidence, such as another person’s verbal testimony about an event or concept (Harris and Koenig, 2006; Harris and Corriveau, 2014). Specifically, with respect to balance principles, research so far has used both primary evidence (i.e., direct experience of anomalous evidence) (Bonawitz et al., 2012) and secondary evidence (i.e., indirect experience of anomalous evidence through a picture book) (Ganea et al., 2017) showing that children can learn from both. In this study we directly compare primary and secondary sources of evidence to examine their relative effectiveness in inducing belief revision about balance in 5-year-old children.

### Current Study

The current study directly compares primary and secondary sources of evidence to determine whether or not there are differences in children’s ability to learn from them. Each source contained both anomalous evidence and mechanistic explanations. We presented the anomalous evidence through either guided activities (Primary Evidence condition), or through examples described and illustrated in picture books (Secondary Evidence condition). Mechanistic explanations were delivered either verbally by the experimenter during the activity (Primary Evidence condition) or included in the information in the picture books (Secondary Evidence condition). We expected that both conditions will improve from pre- to post-test, but we predicted that the primary evidence condition will see more improvement, based on prior research that children prefer and consider primary evidence to be more credible than secondary evidence (Sandoval and Çam, 2011; Koerber et al., 2017).

We developed our pre- and post-test to combine elements from the tasks of Karmiloff-Smith and Inhelder (1974) and Siegler (1976). First, we asked participants to place four different object pairs (two each of equal and unequal weights) on a beam so that the beam would stay perfectly balanced in the air. This placement element is modeled after Karmiloff-Smith and Inhelder’s task, however, we used separate weights to simplify the task and allow for a greater variety of answers. In addition, after children made each placement, we asked them to explain their choices. The use of separate weights and explanations was drawn from Siegler’s task. By combining aspects of both tasks for our test phase, we aimed to explore the possibility of intermediate levels of balance understanding.

We examined 5-year-olds’s understanding because this is an age for which prior research has provided inconsistent evidence. Five-year-olds were categorized as lacking a theory of balance when tested with the placement task (e.g., Bonawitz et al., 2012; Ganea et al., 2017), but as being able to understand weight as a relevant variable for balance when tested with the prediction task (Siegler and Chen, 1998). Further, Siegler and Chen (1998) found that over the course of several trials, 5-year-old children began to accurately predict balance relationships that were affected by distance. Although children were not able to explain their predictions, they reduced their references to weight. In contrast, 4-year-olds did not improve their predictions and continued to reference weight in their explanations (Siegler and Chen, 1998). Further work by Pine et al. (1999) also found improvement in children’s understanding of how to balance asymmetrical blocks in their sample of 5-to-7-year-olds, using a combination of guidance and explanations. This suggests that with support and appropriate evidence, 5-year-olds may be able to move beyond a reliance on weight to understand balance and incorporate distance as another causal dimension.

## Materials and Methods

### Participants

One hundred and two 5-year-olds (*M* = 5.49; range: 5.02—5.99, 51 females, 51 males) participated in this study. Twenty-two additional children were excluded because they had a perfect score on the pretest (*n* = 15), had a language barrier (*n* = 4), or due to parental interference (*n* = 1) or experimenter error (*n* = 2).

Children were randomly assigned to one of three conditions: Primary Evidence (*M*_age_ = 5.51), Secondary Evidence (*M*_age_ = 5.49), and Control (*M*_age_ = 5.49). Each condition had 34 children. For our evidentiary conditions, we developed two different hands-on activities and two different books designed to teach children about balance. The use of two different tasks and books allowed us to ensure that children’s learning is not tied to a specific type of stimulus. Within each condition, children completed *one* activity or read *one* book. The books and activities were matched in the presentation of anomalous evidence and the amount of conceptual information provided to children. The control condition differed in that children either read an unrelated book or were offered the materials from the hands-on activities to explore independently.

Participants were recruited from a database of families who have expressed interest in participating in research. Children were individually tested by a female experimenter in a quiet room at a University laboratory. The sample of children came from a diverse background, including Asian (13%), Aboriginal (1%), Black (3%), White (47%), and Mixed Race (30%) children. An additional 6% of families declined to disclose ethnicity information. Of the families who disclosed this information the majority of children came from middle- and upper-class families. However, 6% of families declined to disclose this information. The mode parental education level was a Bachelor’s Degree, with 5% of families declining to disclose the education for either parent.

### Materials

#### Interventions

In the experimental conditions, children were shown one example of how to balance objects with the same weight, and three examples of how to balance objects with different weight from each source of evidence.

##### Primary evidence

**FIGURE 1:**
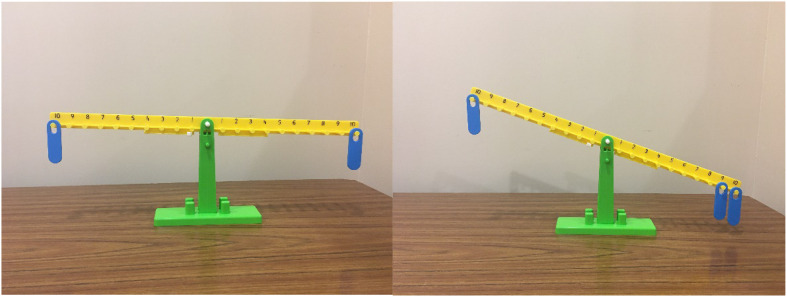
Stimuli for placement activity. Examples of balanced beam with same number of weights on same hook and unbalanced beam with more weights on one side.

**FIGURE 2:**
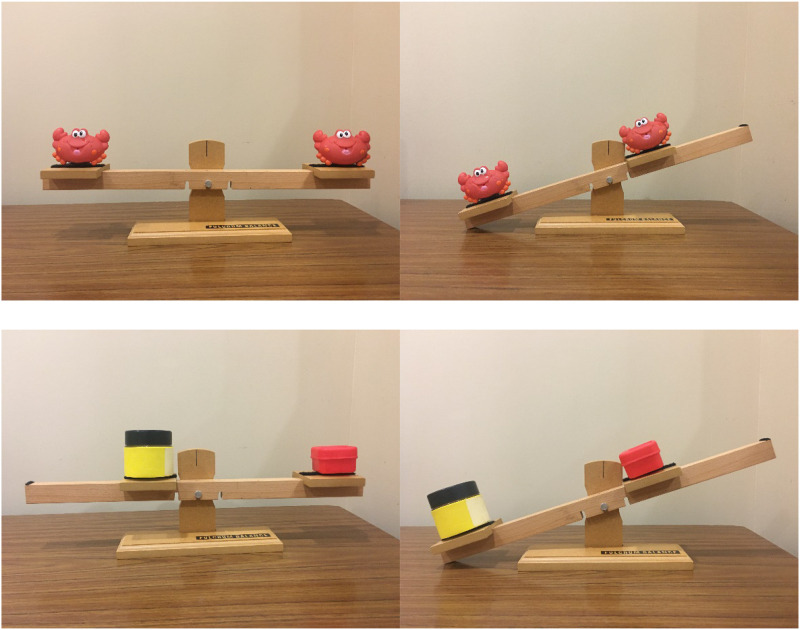
Stimuli for prediction activity. Examples of a balanced beam with same weight objects (crabs) the same distance apart (top left image) and differently weight objects (boxes) with the heavier box closer to the middle (lower left image). Examples of an unbalanced beam with same weight objects (crabs) at different distances (top right image) and differently weight objects (boxes) with the heavier object further from the middle (lower right image).

In the first activity (Placement), children were provided with a beam with different hooks along the beam and different weights that could hang on the hooks. Children hung different weights along the beam to try and balance it (see Figure 1). For the second activity (Prediction), children were given a beam with sides that slid closer to and further from the fulcrum. They were also provided with toy crabs, of the same weight, and marbles, jars, and toys of different weights (see Figure 2). For this activity children were also given a worksheet to record their predictions and results (the activity scripts can be found in Supplementary Appendix A).

##### Secondary evidence

We wrote and illustrated two texts. The first text, a narrative book, had two young children playing at the park on a seesaw. In the book the children balanced one pair of same weight objects, and three pairs of different weight objects on the seesaw. The second book had the same examples but presented in a straight-forward information text and used photographs instead of pictures (illustrations of the narrative book can be found in Supplementary Appendix B; the text from the non-fiction book can be found in Supplementary Appendix B, the photographs from the non-fiction book are not included due to reproduction permissions).

#### Test Phase

**FIGURE 3:**
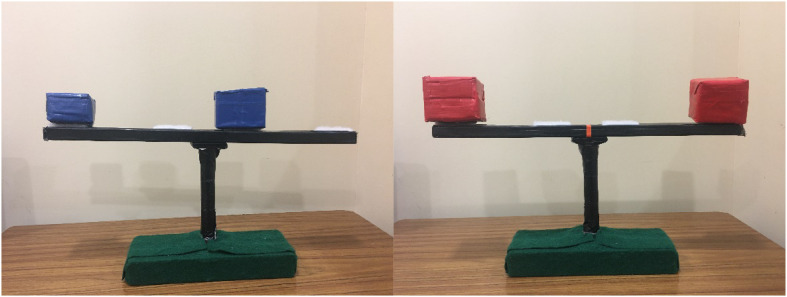
Stimuli for test phase. Correct placement for different weight objects (blue blocks) and same weight objects (red blocks) shown. Note that same weight objects could also both be placed close to the middle.

As a measure of learning, pre- and post-tests were administered. The materials for each test phase included a stand and a beam with four velcro pieces on it. These velcro pieces were placed with two on either side of the middle, one close to the middle and one further from the middle. We also used four pairs of objects in each test phase, for a total of eight object pairs. All of the objects had pieces of velcro on the bottom, corresponding to the velcro on the beam. Within each set of four pairs, two pairs were the same size and weight, and two pairs were different sizes and weights (see Figure 3). For each test phase children received a different object set, but the order in which children received these sets were counterbalanced.

### Procedure

**FIGURE 4:**
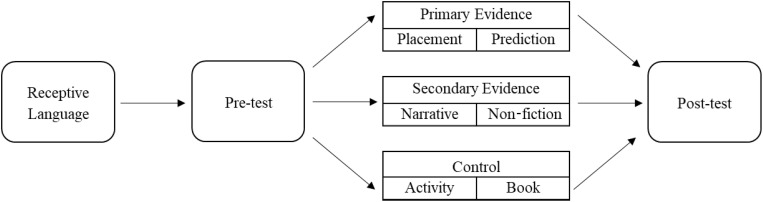
Schematic of study design.

This study used a between-subjects design. Each condition followed the same four-phase protocol, where children were randomly assigned to one of three conditions: Primary Evidence, Secondary Evidence, or Control (see Figure 4). Within each condition, children were randomly assigned to complete one activity or read one book. The entire session was video-recorded and lasted approximately 15–20 min.

#### Receptive Vocabulary

To ensure that children were able to understand the tasks and the conceptual information provided in the intervention, we administered the Toolbox Picture Vocabulary Test (TPVT). The TPVT measures receptive vocabulary using an adaptive computerized format (National Institutes of Health, 2015).

#### Interventions

##### Primary evidence

In the Primary Evidence condition, children completed one of two different activities: Placement Activity and Prediction Activity.

In the *Placement Activity*, children were provided with a balance beam and several weights. On either side of the beam’s fulcrum there were 10 hooks on which children could hang the weights. Each weight was the same, but more than one weight could be hung on the hook (Figure 1). The experimenter first hung a single weight on the 5th hook and asked the child to hang a single weight to make the beam balance. Once successful, the child was given two weights. The experimenter then hung a single weight on the 8th hook and asked the child to hang their two weights together to make the beam balance. Next, the experimenter took two weights and hung them on the 3rd hook and asked the child to hang a single weight to make the beam balance. Finally, the child was given two weights and allowed to hang them on any hook from five to one (inclusive). Together, the child and experimenter decided where to hang the experimenter’s single weight to balance the beam. In each example, the child kept experimenting until they were successful in their placements. The experimenter highlighted that the same weight objects balanced when they were on the same number, and the different weight objects balanced when the heavier ones were closer to the fulcrum and the lighter one was further away.

In the *Prediction Activity* children were provided with a different balance beam. This beam had sliding end seats on it, so that objects could be placed on these seats and then moved closer to or further from the fulcrum (Figure 2). Children were also provided with a worksheet, and told they were going to make predictions about how objects balance on a beam and they would record their predictions and the results on this sheet, just like a scientist does. Children were first given two toy crabs with the same weight. They put these crabs on the seats of the beam and moved the seats until they were able to balance the beam. They were then provided with two marbles that were different weights. They again placed the marbles on the seats and moved them until they were able to balance. This was repeated with two more object pairs that had different weights: jars and toys. As with the Placement Activity, the experimenter highlighted that the same weight objects balanced when they were on the same number, and the different weight objects balanced when the heavier ones were closer to the fulcrum and the lighter one was further away.

For both activities, the intervention ended with the experimenter providing an explanation about why the objects balanced at different places on the beam. The experimenter explained that objects that have the same weight have the same force on the beam, and therefore they balance the same distance away from the fulcrum. In contrast, when objects have different weight, the heavier object has more force than the lighter one, so the heavier object must be placed closer to the fulcrum, and the lighter object must be placed further away, to compensate for this difference in force.

##### Secondary evidence

In the Secondary Evidence condition, children were read one of two different picture books, a Narrative Book or a Non-fiction Book (see Supplementary Appendix B).

The *Narrative Book* told a story of two children, Alice and Luke, playing on a seesaw. As they are playing Luke wonders why he goes up into the air before Alice. Using experiments with buckets of sand, Alice first demonstrates to Luke how two objects that have the same weight need to be the same distance away from the fulcrum in order to balance. She then uses the buckets to demonstrate that once they have different weights, the heavier bucket needs to be closer to the fulcrum in order to balance. She also demonstrates this with rocks of different weight, and finally with the children themselves. At the end of the book Alice provides Luke with an explanation about why the objects balanced at different places on the beam. This explanation was matched with the explanation the experimenter provided to children who completed the primary evidence activities.

The *Non-fiction Book* matched the *Narrative Book* in terms of the examples and information provided to children. The only difference was that information was presented in an expository format as opposed to embedded in a plot.

#### Control

In the Control condition, half the children completed a hands-on activity (Activity Control) and the other half were read a picture book (Book Control).

##### Activity control

In the Activity Control children were provided with all the same materials as the children who completed the Prediction Activity. They were shown how the beam worked and how the seats moved, but were given no further direction with what to do with the beam or the objects, nor any feedback or information about how or why objects balance. The goal of this control was to ensure that mere exposure to the beam was not sufficient for children to learn about balance.

##### Book control

In the Book Control children were read an unrelated picture book about plants (The Tiny Seed, by Eric Carle). The goal of this control was to ensure that exposure to the pre-test was not sufficient for children to make gains in their understanding on the post-test, due to the parallel structure of these tests of learning.

#### Test Phase

Pre- and post-tests were administered using the same procedure but with different object sets. Each test phase consisted of four trials, with each trial using one set of four pairs of objects. Each set of four object pairs consisted of two pairs with the same size and weight, and two pairs with different sizes and different weights. In the different pairs, the bigger object was also always the heavier object. Object sets were counterbalanced between test phases, but due to the possibility of differences in response patterns, within each set the pairs were presented in the same order to participants (same weight, different weight, same weight, and different weight).

During both test phases, participants were first shown a stand and a beam. The experimenter oriented the children to these materials by saying “When I put my beam on my stand like this, it’s like a seesaw or a scale. It can stay perfectly balanced in the air, or one side can go up and one side can go down.” This served both to introduce the beam to children and highlight the mechanics to children, as well as connect the beam to the intervention phase with the term seesaw (from the books) or scale (from the activities). After this introduction, the experimenter told children “Now, I’m going to show you some blocks and ask you some questions about where we can put the blocks on the beam so that it stays perfectly balanced with both sides in the air.” The experimenter also explained to children that they had to put one block on either side of the middle, and demonstrated that on each side, blocks could be placed on two possible velcro strips: one that was closer to the middle, and one that was further from the middle.

Following this orientation children were shown the object pairs one at a time for a total of four trials. For each object pair, the experimenter highlighted that they were either the same size and weight, or different sizes and different weights. Children were asked to place each object pair on the beam so that the beam would stay perfectly balanced in the air. After children placed the objects on the beam, the experimenter placed the beam on the stand, holding it in place so that it would balance regardless of where children had placed the blocks. The experimenter then confirmed with the child “It will balance like this?” If the child answered in the affirmative, the experimenter followed this question with “Why do you think it will balance like this?” Children’s explanations were recorded. If the child did not agree it would balance, they were asked to move the blocks to a space where it would balance, and then were asked to provide an explanation for their placements. The experimenter did not let go of the beam, and therefore children could not see if their placement was correct. Children received neutral feedback (“Thank you”) after answering each test question.

#### Coding

Children’s placement of the blocks at pre-test and post-test were coded by research assistants using video recordings of the sessions. A visual depiction of the correct placement of the blocks can be found in Figure 3. To fit with analyses from previous research, based on these placements, children were categorized into “center theorists” or “mass theorists” or “no theory.” Children who had correct placement of all blocks were coded as “mass theorists,” and children with completely random placement were coded as “no theorists.” Due to the adapted design of our task, we were also able to identify an additional category. We split the center theorists into two groups: “traditional center theorists” and “transition theorists,” Traditional center theorists were those that placed all blocks equal distance from the center, regardless of their weight. Transition theorists were those who had separate theories for same weight object pairs and different weight object pairs, though one theory was incorrect; they either placed different weight pairs both close to the center, and same weight object pairs both far from the center (or vice versa), or they placed same weight object pairs correctly, and different weight object pairs with the light object closer to the fulcrum and the heavier object further away. These children were considered Transition as they understood that same weight and different weight objects would need to be balanced differently, but they did not yet have full understanding of the mass theory. This aligns with the rules proposed by Siegler (1976), which specified that children would focus attention on different variables for same weight and different weight object pairs (Rule 2), prior to moving into an understanding of the interplay between distance and weight (Rule 3, or Mass Theorists). Children who placed the same weight pairs correctly and one different weight pair correctly were also coded as transitional theorists.

We also analyzed the justifications children gave when asked to explain their choices for the placements. The explanations were used as a secondary measure of learning and coded on a scale from 0 to 2. A breakdown of the coding scheme for explanations can be found in Table 1.

**TABLE 1:** Coding scheme for children’s explanations of their balance placement predictions at pre- and post-test.

Score	Placement	Reason	Example
2	Correct	Referencing distance from fulcrum.	Same weight objects: Because they are both far away from the middle. Different weight objects: Because the heavy one it close to the middle.
1	Correct	Referencing distance from fulcrum in conjunction with incorrect information. Reasons that refer to object weight but not distance.	Because they are both far away from the middle and squishy. Because one is heavy and one is light.
0	Correct	Referencing irrelevant information as a justification.	Because they are both square shaped.
0	Incorrect		

Explanation scores were summed across the four trials, such that scores ranged from 0 to 8 for each test phase. Two research assistants coded 100% of the children’s responses. The coders were blind to the hypotheses, test phase and condition of the study. There was high inter-rater reliability determined by Cohen’s κ = 0.82, *p* < 0.001, an 89% agreement rate. The two coders resolved disagreements through discussion.

## Results

We conducted two main analyses of the data. First, we examined how children’s theories changed from pre- to post-test. Second, we examined children’s belief revision as a function of evidence sources from pre- to post-test. Preliminary analyses indicated the two types of books and activities used did not influence the post-test measures. The classification of children as theorists at post-test did not differ by book type, χ^2^(3, *N* = 34) = 2.62, *p* = 0.46 or activity type, χ^2^(3, *N* = 34) = 1.44, *p* = 0.61. Similarly, explanation scores at post-test did not differ by book type, *t*(32) = 0.82, *p* = 0.42, or activity type, *t*(32) = 30.29, *p* = 0.92. As a result we collapsed both types of books and activities in the following analyses.

### Theories

Table 2 shows the number of participants categorized as a type of theorist at pre- and post-test across conditions. Chi-Square goodness-of-fit tests were conducted both at pre-test and post-test to examine whether the observed frequency of theorists was different than chance (all 4 categories were treated as equal, chance level = 25.5, 102/4). The observed frequency of theorists differed from chance both before, χ^2^(3, *N* = 102) = 9.92, *p* = 0.02, and after, χ^2^(3, *N* = 102) = 41.84, *p* < 0.001, the intervention. Table 2 shows that at pre-test 26% of children (26/102) were categorized as no theory, 28% (28/102) as traditional theorists and 34% (35/102) as Transition theorists, but only 13% (13/102) were mass theorists. In contrast, at post-test a smaller percentage of children were categorized as having no theory (17%, 17/102) or traditional theorists (7%, 7/102), and a greater percentage of children were Transition theorists (50%, 51/102) or mass theorists (27%, 27/102).

**TABLE 2:** Frequencies of children who held a certain balance theory as a function of test-phase and condition.

Condition	Pre-theory	Post-theory				Total
		No theory	Traditional	Transition	Mass	
Primary	No theory	1	0	5	1	7
evidence	Traditional	1	1	7	3	12
	Transition	1	0	4	5	10
	Mass	0	0	2	3	5
	**Total**	**3**	**1**	**18**	**12**	**34**
Secondary	No theory	1	1	5	1	8
evidence	Traditional	1	1	2	3	7
	Transition	1	0	9	7	17
	Mass	0	0	1	1	2
	**Total**	**3**	**2**	**17**	**12**	**34**
Control	No theory	4	1	5	1	11
	Traditional	2	1	6	0	9
	Transition	4	2	2	0	8
	Mass	1	0	3	2	6
	**Total**	**11**	**4**	**16**	**3**	**34**
Total	No theory	6	2	15	3	26
	Traditional	4	3	15	12	28
	Transition	6	2	15	12	35
	Mass	1	0	6	6	13
	**Total**	**17**	**7**	**51**	**27**	**102**

A multiway frequency analysis (MFA) was used to investigate the relative importance of pre-test, post-test and condition in predicting the expected frequencies, while maintaining adequate fit between expected and observed frequencies. MFA is an extension of the chi-square goodness-of-fit technique, where the goal is to create a model that accounts for the observed and expected frequencies being equal (a non-significant chi-square statistic) while using the fewest variables as possible in the model (Agresti, 2002; Howell, 2007; Tabachnick and Fidell, 2012). MFA is a non-parametric statistical procedure for discrete variables with two or more levels. This type of analysis was selected to accommodate the frequency nature of the dependent variable, and the presence of within-subject factor (pre- and post-test scores) while allowing for the analysis of main effects and interactions (Vokey, 2003).

We generated three preliminary crosstabs comparing pre-test to post-test and each test phase separately by condition to ensure that each cell had sufficient frequencies to conduct an MFA. The crosstabs indicated that no cell had an expected frequency of 0, but 14 of the 40 cells (35%) had frequencies less than 5. Collapsing categories is one way to deal with this violation that no more than 20% of the cells should have expected frequencies below 5 in order to retain appropriate power (Agresti, 2002; Tabachnick and Fidell, 2012). We decided to retain all levels of variables in the tested model, despite the potential loss of statistical power (Wickens, 1989), because all categories were of interest. A *post hoc* power analysis was conducted, revealing that for a calculated effect size ω = 0.39, power was sufficient for this study (1 − β) = 0.80.

Using a backward hierarchical approach to model building, a saturated log-linear model with all one-way, two-way, and three-way interactions was examined. The likelihood-ratio (*LR*) χ^2^ values for the overall effects found that the two-way interaction between Condition × Post-theories and the one-way effects of Pre- and Post-theories achieved significance (Table 3). Backward hierarchical solution statistics identified a model with the two-way Condition × Post-theories interaction and one-way Pre-theories main effect as best fitting, LR χ^2^(*df* = 33) = 33.32, *p* = 0.45. The non-significant LR χ^2^ value means that the model has the smallest number of effects that yields an adequate fit between the expected and observed frequencies. A custom model using all significant effects was also assessed and found to be not significant, LR χ^2^(*df* = 33) = 31.74, *p* = 0.53. As both tests were non-significant, we can conclude that the associations dropped from the saturated model were not needed to explain the distribution of frequency data. A visual inspection of plotted residuals confirmed that the observed standardized residuals were acceptably close to those that were expected.

**TABLE 3:** Multiway frequency analysis K-way effects and partial associations.

Effect	Partial χ^2^	df	*p*-value	Number of iterations
Condition	0.00	2	>0.99	2
Pre-test	10.90*	3	0.01	2
Post-test	41.90*	3	<0.001	2
Condition × Pre-test	9.90	6	0.13	2
Condition × Post-test	17.95*	6	0.006	4
Pre-test × Post-test	11.66	9	0.23	4

*p < 0.05.

The two-way association between condition and post-theories significantly predicted the expected frequencies. This fits our hypothesis, as we expected that the experimental conditions will change children’s theories compared to the control conditions. From Table 2, we see 9 and 11 children from the primary and secondary conditions made Mass Theorist predictions after the intervention, compared to only one child in the control condition. We also observed a greater number of children in the control condition retaining their theory or reverting back to random predictions. The presence of one-way effects suggested the independence of the variables but should not be interpreted if nested in higher order associations (Agresti, 2002). Therefore, we focused exclusively on the pre-theories which was an important predictor of the expected frequencies above and beyond the other effects. The majority of children shifted to a higher ranked theory with only 19 out of 102 children regressing. At post-test, most children were categorized as transition (*n* = 51) and mass (*n* = 27) theorists. Further, most children who were categorized as mass theorists at post-test were traditional and transition theorists at pre-test. This indicates that children’s pre-theories affected whether children revised or retained their current theory after the intervention.

### Explanations

Preliminary analyses for explanations showed no effect of age, gender or TPVT, therefore these variables were not considered in the following analyses. A one-way ANOVA also revealed that pre-test explanation scores were similar across all three conditions at baseline, *F*(2,99) = 1.50, *p* = 0.23, $\eta_{\text{p}}^{2}$ = 0.03.

**FIGURE 5:**
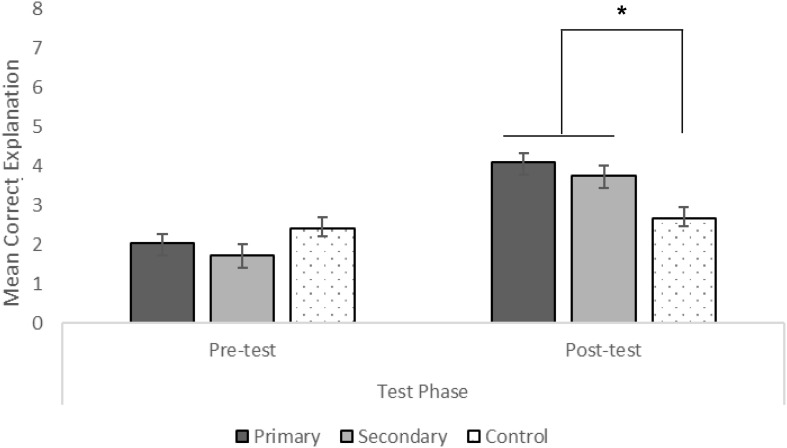
Mean explanation responses out of 8 as a function of test phase and condition (^∗^*p* < 0.001).

A 2 (test phase) × 3 (condition) mixed-measures ANOVA showed a significant main effect of test phase, *F*(1,99) = 78.47, *p* < 0.001, $\eta_{\text{p}}^{2}$ = 0.44. Pairwise comparisons with Bonferroni corrections indicated that the mean difference scores at post-test were significantly higher than scores at pre-test (*p* < 0.001, 95% CI = 1.11, 1.80). There was no main effect of condition, *F*(2, 99) = 1.52, *p* = 0.220, $\eta_{\text{p}}^{2}$ = 0.03. However, there was a significant interaction between test phase and condition, *F*(2,99) = 13.24, *p* < 0.001, $\eta_{\text{p}}^{2}$ = 0.21. We used estimated marginal means with Bonferroni corrections to determine the nature of this interaction (see Figure 5). There was a significant change in children’s explanation scores from pre-test to post-test in the primary (*p* < 0.001, 95% CI = 1.54, 2.70) and secondary (*p* < 0.001, 95% CI = 1.48, 2.63) evidence interventions but no significant change for the control condition (*p* = 0.38, 95% CI = −0.31, 0.84). In terms of condition differences at post-test, children in the primary (*p* < 0.001, 95% CI = 0.52, 2.48) and secondary (*p* = 0.02, 95% CI = 0.17, 2.13) evidence conditions had explanations that were significantly higher than those of children in the control condition. There were no differences between children’s explanations in the experimental evidence conditions at post-test (*p* > 0.99, 95% CI = −1.33, 0.63).

## Discussion

This study evaluated the impact of primary and secondary sources of evidence on children’s theories about balance relations. There were two main findings in this research. First, children’s prior theories played an important role in determining whether children maintained or modified their beliefs about balance in response to the intervention. Second, children were able to learn that distance plays a crucial role in balancing asymmetrical objects when they received mechanistic explanations combined with either primary or secondary anomalous evidence.

The current findings are consistent with previous research in two ways. First, young children develop theories about how to balance different types of objects through their informal daily experiences (Karmiloff-Smith and Inhelder, 1974; Siegler and Chen, 1998). Most children had an intuitive theory about how to balance symmetrical and asymmetrical objects prior to the intervention, with only 25% (26 out of 102) of children categorized as having no theory. Second, a greater number of children from the experimental conditions advanced to a mass theory after the intervention compared to the control condition, particularly when they possessed a theory, either a traditional or transition theory. Karmiloff-Smith and Inhelder (1974), showed that the presence of a theory aided children in exploring how to balance asymmetrical beams. Similarly, when given the chance to balance objects over several days, possessing a theory prior to beginning the task promoted the advancement through Siegler’s (1976) four rules about balance relations (Siegler and Chen, 1998). The authors postulated that the 4- and 5-year-olds who had a theory appreciated the value of systematicity and were more likely to form and follow more advanced rules than children who did not have a theory.

The current research unifies previous research on children’s balance theories. Previous research showed that, with time or explicit instructions, 5-year-olds can consider the effect of distance when balancing asymmetrical blocks (Siegler and Chen, 1998; Pine et al., 1999) whilst other studies found that when 5-year-olds witnessed evidence without an explanation they were not able to learn how to balance asymmetrical blocks (Bonawitz et al., 2012; Ganea et al., 2017). Our finding of an intermediate theoretical phase, Transition Theorists, is important in resolving these divergent findings. Specifically, we found that at post-test, 50% of children were categorized as Transition Theorists, demonstrating the prominence of the Transition phase stage. Further, many children who were Traditional Theorists or did not have a theory at pre-test were categorized as Transition Theorists at post-test. As such, the inclusion of this phase better captures the progression of 5-years-old ability to form theories on how to balance unevenly weighted blocks. We found significant learning for this younger age group, with similar degrees of learning from both primary and secondary sources of evidence, which indicates that 5-year-olds can develop theories about balance that incorporate distance when tested immediately following explicit instruction.

These findings also showed that credible sources of evidence that provide anomalous examples combined with explanation benefit belief revision. Our experimental conditions were structured so that each participant received the same number of examples and the connections between the examples and the explanation were matched. Multiple exemplars of anomalous evidence can promote deeper understanding of the target concept (Gentner, 1983, 1989) by reducing invalid inferences (Vosniadou and Skopeliti, 2019) as well as aiding in visualizing the explanation (Vosniadou, 1989; Brown, 1992, 1993). Further, the explanations not only highlighted the significance of a novel variable (i.e., distance), which is challenging for younger children to consider on their own, but it also highlighted its causal role (Siegler and Chen, 1998). This work supports Brown’s (1992) conclusion that examples must be clearly analogous to the concept in question and that they be presented in a connected sequence referencing an explicit mechanistic model. In other words, the structure of our experimental conditions, with clearly analogous anomalous evidence situated within a causal explanatory framework, was the probable mechanism that facilitated the creation and revision of theories for younger children.

While the current work shows that children learn regardless of the source of evidence, this domain is not one with longstanding prior beliefs. That is, by early elementary school most children have progressed from a center theorist to a mass theorist (Karmiloff-Smith and Inhelder, 1974). This can be contrasted with other domains where naive beliefs persist into adulthood, such as the belief that heavier objects fall faster than light objects (Kavanagh and Sneider, 2007). Children’s belief revision is likely influenced by the interaction between the strength of their prior beliefs and the complexity of the evidence presented. Children may have markedly diverse responses to different sources of evidence across various knowledge domains. An open question remains whether children would benefit from multiple exemplars alone, or if the explanations provided an additive benefit, especially when children have strong competing prior beliefs. Further research is needed to explore children’s responses to different sources of evidence about various scientific concepts.

Additionally, our study was a single intervention completed in a one-on-one setting. Proceeding from belief revision to conceptual change is a gradual process (Vosniadou, 2002) as new beliefs do not simply replace old beliefs. Prior conceptions continue to coexist with newly learned information where either may be utilized depending on the situation (Shtulman and Valcarcel, 2012; Shtulman and Lombrozo, 2016). Given the study design we cannot discern if children maintain their learning at a delay. Therefore, it is uncertain whether children underwent conceptual change or short-term belief revision. More research is needed to examine these results over a longer period of time. Future work should also look at how these questions transfer to a naturalistic setting, to determine the implications for early science education.

## Conclusion

This research adds to our understanding of how children develop scientific theories and the incremental changes that these theories undergo as children are exposed to anomalous evidence and causal explanations. With increased exposure to reliable sources of evidence and accompanying explanations, children can reach an understanding of scientific principles, at least in the domain of balance, earlier than previously thought (Karmiloff-Smith and Inhelder, 1974). Such findings justify the call for science education in the early years (Bowman et al., 2001; Gelman and Brenneman, 2004; Eshach and Fried, 2005; Duschl et al., 2007; Morgan et al., 2016). Promoting an earlier understanding of these concepts will serve as a foundation for more advanced concepts as children progress through school (Hardy et al., 2006). Indeed, scientific knowledge at preschool predicts children’s science achievement in later grades (Morgan et al., 2016). Further research on how children’s scientific understanding develops and what approaches improve their understanding can help conceptualize how to build effective early science education.

## Data Availability Statement

The data available on the Open Science Framework website: https://osf.io/84xap/?view_only=6747d7eec0054c5aa22951a8a0b95101.

## Ethics Statement

The studies involving human participants were reviewed and approved by University of Toronto Ethics Board. Written informed consent to participate in this study was provided by the participants’ legal guardian/next of kin.

## Author Contributions

PG: conceptualization, methodology, writing – review and editing, supervision, and funding acquisition. NL and VV: conceptualization, methodology, investigation, analysis, and writing – original draft. All authors contributed to the article and approved the submitted version.

## Conflict of Interest

The authors declare that the research was conducted in the absence of any commercial or financial relationships that could be construed as a potential conflict of interest.
