# Supplementary material for: Prompting Children’s Belief Revision About Balance Through Primary and Secondary Sources of Evidence
Source: Front Psychol. 2020 Jul 22;11:1503. doi: 10.3389/fpsyg.2020.01503 (PMC7387500; doi:10.3389/fpsyg.2020.01503)
Supplement: Supplementary file 1 [file Presentation_1.pdf]

Appendix A: Activity Scripts

Placement Activity

Have you ever played on a seesaw at the park? Usually when two people sit down on a seesaw at the same time, one side goes up into the air.

Have you wondered why one person goes into the air right away even if you sit down at the same time?

That's because usually the two people don't weigh the same so the seesaw isn't balanced, so the smaller and lighter person goes up. When we aren't pushing, the heavier side goes to the bottom, and the lighter side goes to the top.

Some people think that objects always balance in the centre, but that's not always what happens.

There is a way to balance a seesaw even if two people or objects don't weight the same.

In this activity, let's use a scale to learn how to balance objects. We will see where we can place the weights on each side of the scale to balance it.

Trial 1: Light/heavy weight closer to the centre

Let's start by placing these weights on the scale to balance it. I am going to put my weight on number 5. Is this your weight the same weight as mine? Yes! Can you place your weight on the scale so that it balances?

When two things that are the same weight are on the scale, it balances when both weights are the same distance away from the middle.

Trial 2: Heavy weight close to the centre

Here is another weight. This time I am going to get two weights, and you are only going to get one. Are my two weights together heavier than your weight or the same? That’s right, my two weights are heavier than your weight. I am going to play my two weights on number 4. Can you place your one weight so that the scale balances? (help child to decide on a number – number 8 will balance)

If there are things with different weights on the scale, you need to place your weights so that the heavier weights are closer to the middle. That will make it balance.

Trial 3: Light object furthest from the centre

Here is another weight. This time I am going to get one weight, and you are only going to get two. Is my one weight lighter than your two weights together or the same? That’s right, your weights are heavier than my one weight. I am going to play my weight on number 6. Can you place your two weights together on the same number so that the scale balances? (help child to decide on a number – number 3 will balance)

When we put these weights on the balance beam, we need to put the weight on so that the lighter weight is further away from the middle. The side with the heavier weights needs to be closer to the middle. The lighter one is on the longer side and the heavier one is on the shorter side. Then it will balance.

Today we learned that things don’t always balance when they are the same distance away from the middle. They only balance when they are the same distance away from the middle if they weight the same.

You can still balance objects even if they don’t weight the same, but they won’t balance in the middle. To balance objects with a heavy and light side, we need to move the object so that the heavier side is closer to the middle and the lighter side is farther away.

Prediction Activity

Have you ever played on a seesaw at the park? Usually when two people sit down on a seesaw at the same time, one side goes up into the air.

Have you wondered why one person goes into the air right away even if you sit down at the same time?

That's because usually the two people don't weigh the same, so the seesaw isn't balanced, so the smaller and lighter person goes up. When we aren't pushing, the heavier side goes to the bottom, and the lighter side goes to the top.

Some people think that objects always balance in the centre, but that's not always what happens.

There is a way to balance a seesaw even if two people or objects don't weight the same.

In this activity, let's use a balance beam to learn how to balance objects. We can see how to move each side of the balance beam with objects on it in order to balance it.

Trial 1: Both objects with the same weights (heavy/light) placed at end

Here are two objects. Are they the same weight? Yes! Is the seesaw balanced? Yes, it is!

When two things that are the same weight are on the balance beam, it balances when both objects are the same distance away from the middle.

Trial 2: Light & Heavy furthest away from the fulcrum

Let's look at these two objects. Are they the same weight? No! Can you move the right/left side of the seesaw to balance the objects?

If incorrect: The seesaw is not balanced! How can we move one side of the seesaw so that it can balance?

If there are things with different weights on the balance beam, you need to move the beam so that the heavier object is closer to the middle. That will make it balance.

Trial 3: Light object on right end and heavy object on left end

Let's look at these two objects. Are they the same weight? No! Can you move the right/left side of the beam to balance the objects?

If incorrect: The seesaw is not balanced! How can we move one side of the beam so that it can balance?

When we put these boxes on the balance beam we need to move the sides so that the seat with the lighter box is further away from the middle. The seat with the bigger box needs to be closer to the middle. The lighter one is one the longer side and the heavier one is on the shorter side. Then it will balance.

Today we learned that things don't always balance when they are the same distance away from the middle. They only balance when they are the same distance away from the middle if they weight the same.

You can still balance objects even if they don't weight the same, but they won't balance in the middle. To balance objects with a heavy and light side, we need to move the object so that the heavier side is closer to the middle and the lighter side is farther away.

Appendix B: Books

Narrative Book

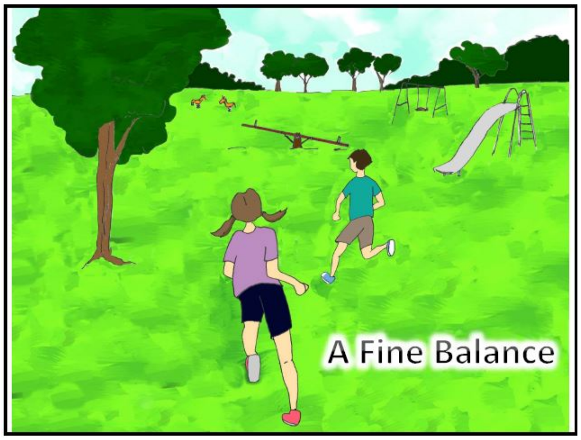
A Fine Balance

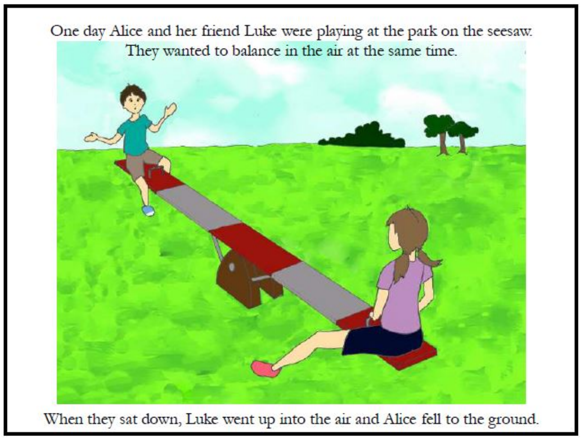
One day Alice and her friend Luke were playing at the park on the seesaw. They wanted to balance in the air at the same time.

When they sat down, Luke went up into the air and Alice fell to the ground.

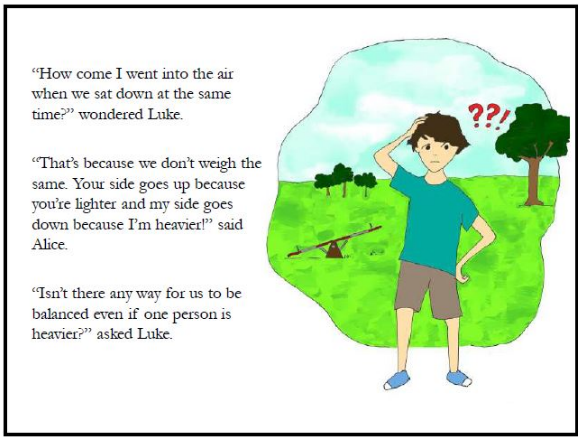
"How come I went into the air when we sat down at the same time?" wondered Luke.

"That's because we don't weigh the same. Your side goes up because you're lighter and my side goes down because I'm heavier!" said Alice.

"Isn't there any way for us to be balanced even if one person is heavier?" asked Luke.

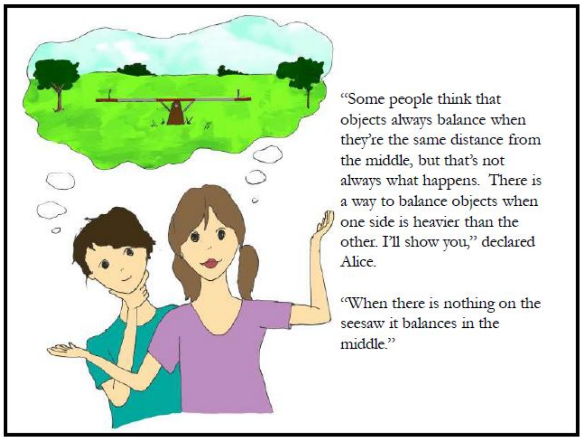
"Some people think that objects always balance when they're the same distance from the middle, but that's not always what happens. There is a way to balance objects when one side is heavier than the other. I'll show you," declared Alice.

"When there is nothing on the seesaw it balances in the middle."

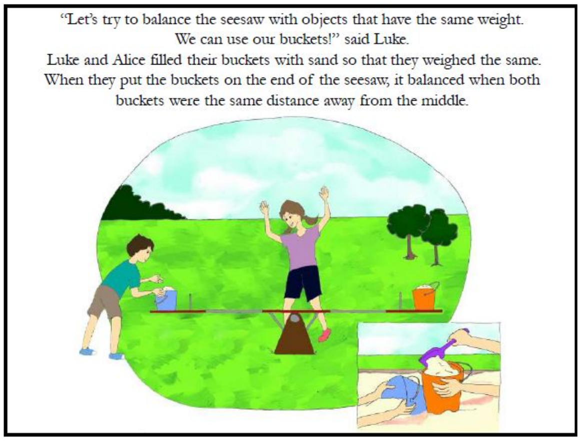
"Let's try to balance the seesaw with objects that have the same weight. We can use our buckets!" said Luke.
Luke and Alice filled their buckets with sand so that they weighed the same. When they put the buckets on the end of the seesaw, it balanced when both buckets were the same distance away from the middle.

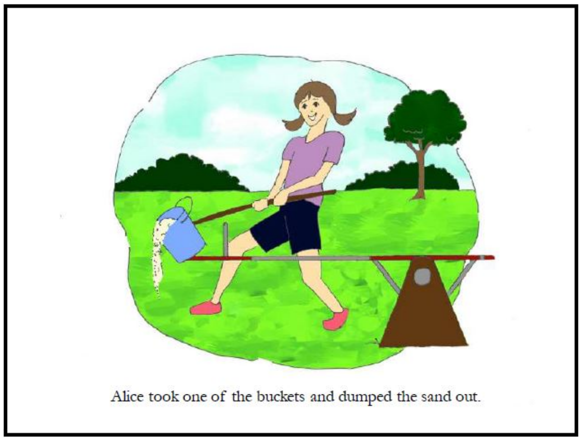
Alice took one of the buckets and dumped the sand out.

When she put the empty bucket back on, the light bucket went up and the heavy bucket dropped down. “Now the buckets don’t weigh the same so it doesn’t balance when they are the same distance away from the middle,” said Luke.

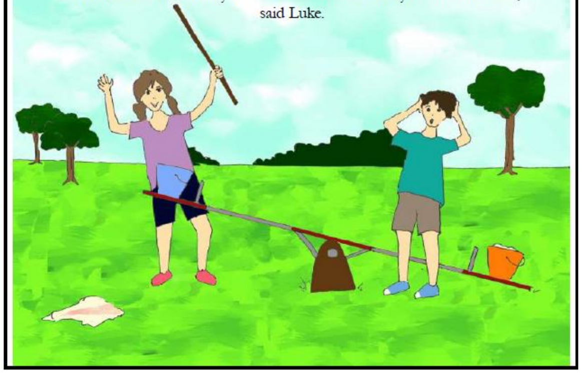

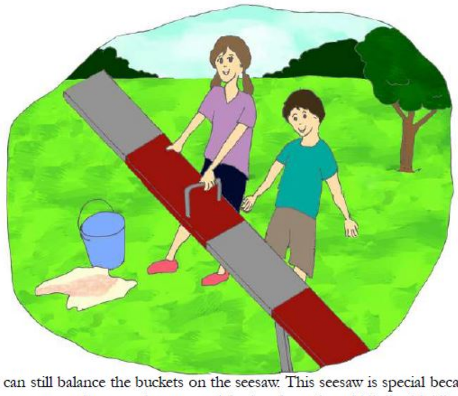

“We can still balance the buckets on the seesaw. This seesaw is special because we can move the seats closer to and further from the middle,” said Alice.

Alice moved the heavier bucket closer to the middle. She moved the seat holding the lighter bucket so that it was farther away. Now the seesaw is balanced.

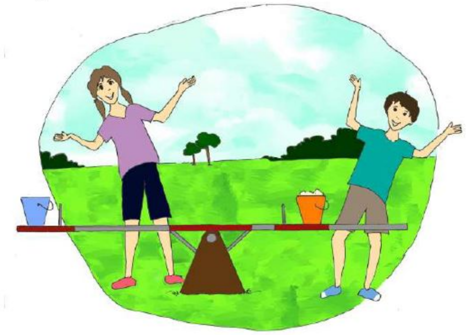

“Do you want to try and make something balance this time?” Alice asked. “Okay, I’ll use rocks!” said Luke. Luke got one heavy rock and one light rock.

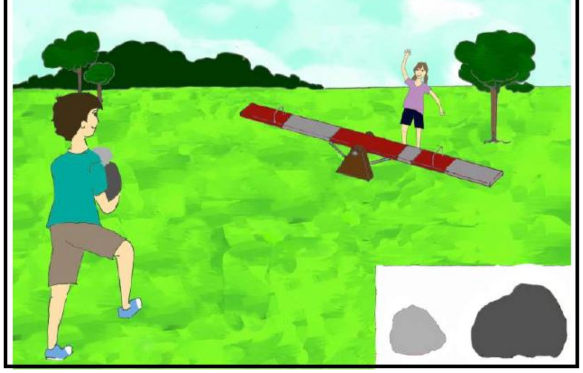

Luke put the heavy rock on the seat that was closer to the middle and put the lighter rock on the seat that was farther away from the middle. Now the seesaw is balanced.

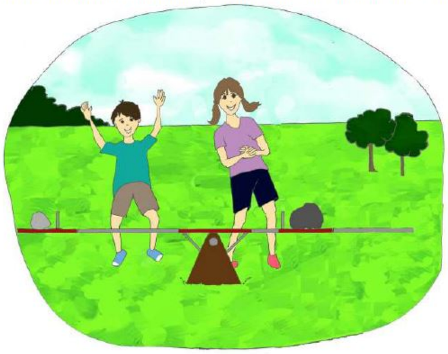

“I know how we can balance ourselves,” said Luke “You are heavier than me so you need to sit on the side that is closer to the middle! I’m lighter, so I need to sit on the side that is farther away.”

When Alice and Luke sat down at the same time, they stayed balanced in the air!

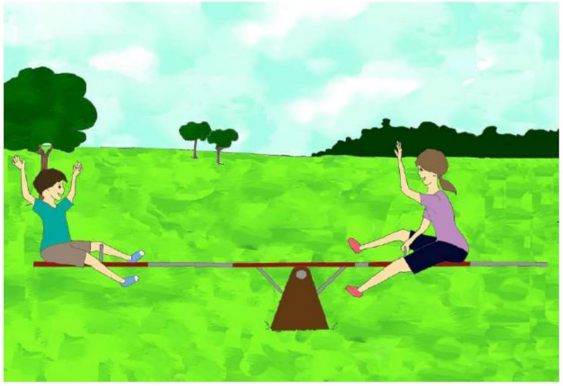

"So what did you learn today?" asked Alice.

"Objects with the same weight balance when they are the same distance from the middle," Luke said.

"Right, a seesaw will only balance in the middle if the two objects have the same weight because then the force on both sides will be the same," Alice explained.

"When you have a heavy and light side, the seesaw won't balance in the middle because the heavier object has more force than the lighter object. In order to make a heavy and light object balance we need to make the force of each side the same," said Alice.

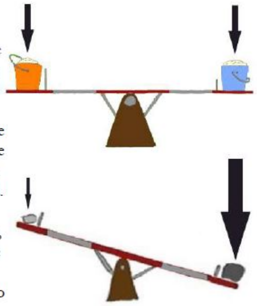

"We make the heavier side have a smaller force by moving the heavier object closer to the middle and the lighter side have a bigger force by moving the light object farther away," explained Alice.

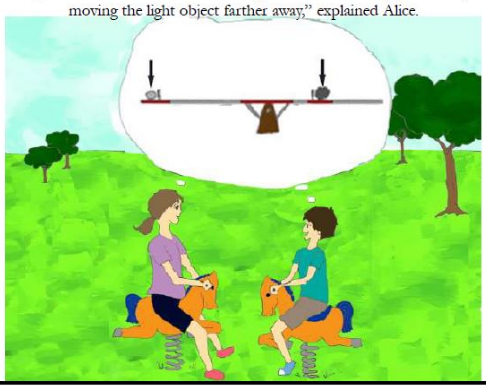

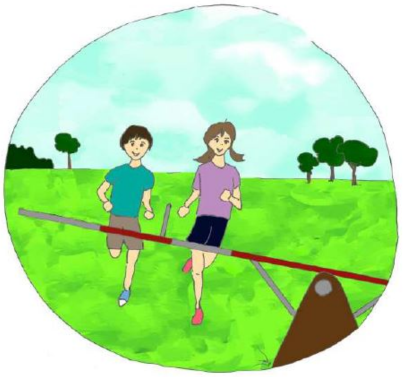

Luke ran to the seesaw. "But sometimes, it's fun to not balance, so I can be up in the air!" he laughed.

Information Book Text

Cover
Balancing Objects

Page 1
Introduction
Have you ever played on a seesaw at the park and wanted two people to balance in the air at the same time? Usually when two people sit down, one side goes up into the air and the other falls to the ground.

Page 2
Have you wondered why one person goes into the air even if you sit down at the same time? That's because two people usually don't weigh the same. The lighter person goes up and the heavier person goes down. Is there a way to balance a seesaw even if two people don't weigh the same?

Page 3
Balancing Seesaws
Some people think that objects always balance when they're the same distance from the middle, but that's not always what happens. There is a way to balance objects when one side is heavier than the other. When there is nothing on the seesaw it balances in the middle.

Page 4
Balancing Objects that Weigh the Same
First, let's balance the seesaw with objects that weigh the same. We can use buckets to test this out! We can get two buckets and fill them with sand so they weigh the same. When we put the buckets on the ends of the seesaw, it balances when both buckets are the same distance away from the middle.

Page 5
Balancing Objects that have Different Weight
Let's take one of the buckets and dump the sand out.

Page 6
When we put the empty bucket back on, this time the light bucket goes up and the side with the heavy bucket drops down. Now the buckets don't weigh the same so it doesn't balance when they're the same distance from the middle.

Page 7

We can still balance the buckets on the seesaw. You need a special seesaw. The seesaw needs to have seats that move closer to and further from the middle.

Page 8
You need to put the heavier bucket on the side that is closer to the middle. You need to move the seat with the lighter bucket so that it is farther away. Now the seesaw will balance.

Page 9
Experimenting with Rocks
You can make seesaws balance with all sorts of things!
Let’s try using rocks. We have one heavy rock and one light rock.

Page 10
We need to put the heavy rock on the seat closer to the middle and put the lighter rock on the seat that is farther away. Then the seesaw will balance.

Page 11
Balancing Two People on a Seesaw
The person who is heavier needs to sit on the side that’s closer to the middle. The lighter person needs to sit on the side that is farther away. So, when the two people sit down at the same time, they will stay balanced in the air!

Page 12
[No text]

Page 13
Conclusion
What did we learn today? Objects with the same weight balance when they are the same distance from the middle.
A seesaw will only balance in the middle if the two objects have the same weight, because then the force on both sides will be the same.
When you have a heavy side and a light side, the seesaw won’t balance when the objects are the same distance from the middle because the heavier object has more force than the lighter one.
In order to make a heavy and a light object balance we need to make the force on both sides the same.

Page 14
We make the heavier side have a smaller force by moving the heavier object closer to the middle.
We might the lighter side have a bigger force by moving the lighter object farther away.

Page 15
But sometimes it’s fun to not balance, so one person can go up in the air!
